# Abstracts from Foreign Exchanges

**Published:** 1886-02

**Authors:** 


### ABSTRACTS FROM FOREIGN EXCHANGES.

BIRNBAUM, of Cologne, gives the history of a woman on whom Cæsarian section was performed the fifth time. All the children were saved, but the woman died from embolism of the pulmonary arteries.

Pettenkofer, of Munich, in a criticism of Cunningham's latest work on cholera gives the author's views in the following summary:

1. Cholera is a disease, which may originate *de novo* anywhere; it is but a severe form of *cholera nostras*.

2. Intercourse of men is of no importance to its spreading. The agent is not adherent to men or their excretions; it is not a living virus. The disease is based on local and atmospheric influences.

3. To protect against cholera, quarantine, isolation or disinfection is fruitless. The only means is assanitation.

Pettenkofer is a bitter enemy of Koch's baccillus theory. As known, he seeks the cause of cholera in certain features of the soil, and if there were any truth in the baccillus, it will have to be brought into line with Pettenkofer's theory as one of the agents.

Klebs, on the other hand, acknowledges the importance of the baccillus, after having witnessed Cecis' experiments. His theory is that the product of the baccilli act as a poison, acting by reflex on the peripheric vaso-motor nerves, causing in this way the status algidus; somewhat like muscarin. His experiments support this view, which is set fourth already by Koch himself.

---

Silbermann (*Deutsche Med. Zeitschft*) pretends to have cured two cases of excessive anæmia by hypodermic injection of blood, a method devised by Ziemssen.

---

Potain (*Revue de Med.*) gives instances of infection by tuberculosis of one part of a married couple from the other. Mostly the wife is the victim. He cites a case reported by Weber, of a sailor, who infected four wives he had married one after the other. Potain considers childbed as predisposing for infection in consequence of its weakening effect upon the system.

---

Laker (*Deuches Archiv*) gives a case in which fifty-nine heads of taenia soleum were found in the fæces of a peasant woman rolled up in a lump. In the following days two more such lumps passed, according to the statement of the patient. The individuals were mostly undeveloped.

---

Mikulicz (*Centralblatt fuer Chirurgie*), after stating the dangers of total extirpation of the thyroid gland (Goitre), namely, cachexia, tetania, epilepsy and paralysis of the laryngeal muscles, gives a new operation, which he performed seven times with perfect results. Even a case of Basedow's (Graves') disease, was perfectly cured. His operation, which he calls resection, consists in loosening the gland in front sufficiently so as to get at the hylus, the place behind, where the nervus recurrens and the vessels are underlying. The superficial and the superior thyroid vessels have to be carefully secured before. The remaining piece takes the part of the pedicle, like in ovariotomy, and is treated like it by cat-gut ligatures and then left alone.

---

Some time ago a new sign of pregnancy was called attention to by Hegar and Reinl. Recently Compes (Berliner Klini: Wochenseh) gives evidence of this sign as absolute certainty. It consists in a softening of the lower uterine portion, which is naturally the thinest part. The difference between this softness and the hardness of the neck is considered characteristic. The procedure is as follows: The finger has to be inserted high np into the rectum; if not easily done, some warm water may be injected. The other hand presses the womb from outside, toward the finger in the rectum, which then can, with ease, palpate the neck and the posterior surface of the womb. The compressibility of the lower uterine segment is thus found.

---

Mikalicz (Wiener Kliniæk) gives a new case of cure of threatening death in acute anemia by infusion of salt water. After speaking of the good results obtained in twelve cases, by different surgeons, he states that transfusion of blood is dangerous, on account of the decomposition of the foreign blood cells causing intoxication by hæmoglobulin. As the danger mostly consists in the subnormal intravascular pressure, and to the consequent ischæmia of the nerve centres, the increase of the quantity of the fluid is the most important feature. The solution best adapted is: Chlorate of soda (table salt) ℥iss, carbonate of soda gr. 15, water about ℥xxx.

The infusion is best done in one of the superficial veins of the arm (of course, centripetally); and any needle or fine trocar, combined with a syringe, will do. Everything has to be disinfected, and the fluid has to be injected very slowly. One litre is a sufficient quantity.

---

Kaegler (*Deutche Med. Zeitsch*) recommends for erysipelas an ointment of resorcin, about ʒiss to vaseline ℥.

---

Lehnhartz (*Charite Annalen*) has tried antipyrine in acute articular rheumatism, and is highly pleased. He recommends it, when salicylic acid has failed, from 15 to 120 grains per day.

---

Very interesting statistics are given in a meeting of the Berlin Gynecological Society regarding malignant ovarian tumors. In the last nine years, among bad ovariotomies performed by Schroeder, there were 100 malignant tumors. This is 64 per cent. On these, 86 operations were finished; on 14, only exploratory incisions were made. Out of the 100, 19 died directly after the operation, 3 after exploratory incisions. Cured of the remainder for longer than one year, 19; all the others died from recidives.

